# Draft Genome Sequences of Lacticaseibacillus rhamnosus cek-R1, Lacticaseibacillus paracasei cek-R2, and Lentilactobacillus otakiensis cek-R3, Isolated from a Beetroot Product

**DOI:** 10.1128/MRA.00921-21

**Published:** 2022-01-06

**Authors:** Eszter Kaszab, Levente Laczkó, Krisztina Bali, Eszter Fidrus, Krisztián Bányai, Gábor Kardos

**Affiliations:** a Veterinary Medical Research Institute, Budapest, Hungary; b Department of Metagenomics, University of Debrecen, Debrecen, Hungary; c MTA-DE “Lendület” Evolutionary Phylogenomics Research Group, Debrecen, Hungary; d Department of Pharmacology and Toxicology, University of Veterinary Medicine, Budapest, Hungary; University of Arizona

## Abstract

Lactic acid bacteria (LAB) participate in fermentation processes and have probiotic potential. The genomes of three LAB strains, Lacticaseibacillus rhamnosus cek-R1, Lacticaseibacillus paracasei subsp. paracasei cek-R2, and Lentilactobacillus otakiensis cek-R3, isolated from a beetroot product, were characterized. The results contribute to our understanding of the beneficial properties of LAB.

## ANNOUNCEMENT

The popularity of vegetarianism/veganism has led to a growing demand for nondairy probiotic products (1). Thus, broadening of the choice of commercially available products with probiotic potential is of high importance (2). Consuming fresh and fermented fruits and vegetables (including legumes) offers beneficial effects, as they are rich in carbohydrates, vitamins, antioxidants, and minerals and free from dairy allergens (1, 2). Probiotic lactic acid bacteria (LAB) help to improve the microbiological and keeping qualities of fermented food products (1, 2). Lacticaseibacillus rhamnosus and Lacticaseibacillus paracasei play an important role in fermentation processes and are among the most significant probiotic organisms (3–5). Until now, Lentilactobacillus otakiensis was described only from a traditional Japanese pickle (6, 7). Identification and characterization of strains found in various food products may help us to understand the background of their probiotic properties.

**FIG 1:**
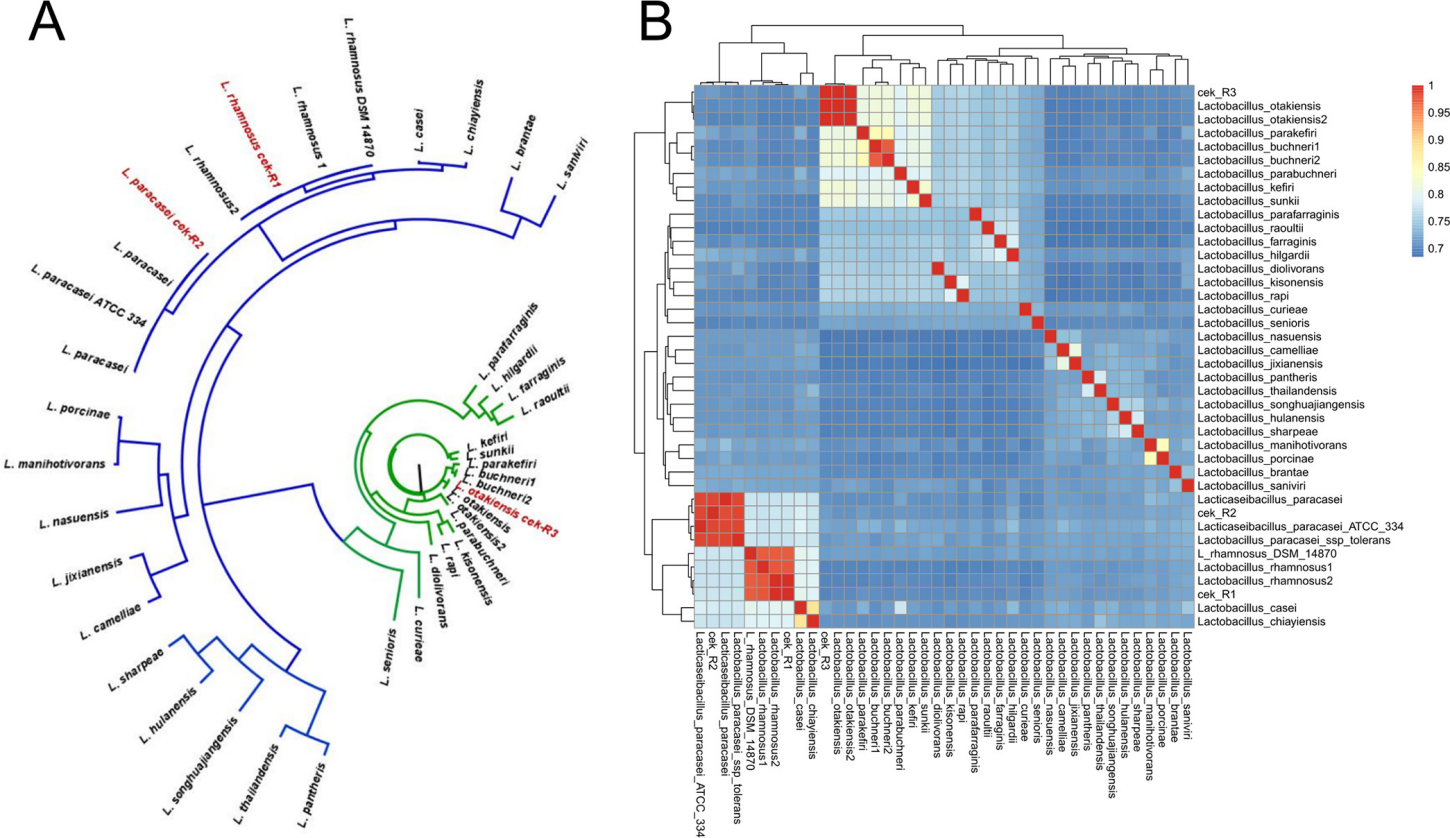
(A) Approximate maximum likelihood phylogenetic tree of core genomes retrieved from representative *Lacticaseibacillus* sp. and *Lentilactobacillus* sp. strains. The phylogenomic analysis is based on the concatenated sequence alignment of protein sequences for the 341 single-copy core genes. All branches had support values of >0.98. The sequences identified in this study are shown in red. The members of *Lacticaseibacillus* are indicated by the blue branches; the *Lentilactobacillus* sp. strains are shown in green. (B) Heat map of the average nucleotide identities (ANIs) of representative *Lacticaseibacillus* sp. and *Lentilactobacillus* sp. strains. The cladogram above and left of the heat map represents the hierarchical clustering of strains as calculated by the pheatmap R package using the pairwise ANI values.

Here, we present the draft genome sequence of L. rhamnosus cek-R1, *L. paracasei* subsp. *paracasei* cek-R2, and *L. otakiensis* cek-R3. A beetroot product (beetroot prepared with sugar and vinegar and seasoned with horseradish) was purchased from a chemical-free farm in Dinnyés, Hungary. The sample was sliced and incubated overnight in brain heart infusion broth (Liofilchem, Italy) at 37°C; then, ∼20 μl was plated onto de Man-Rogosa-Sharpe (MRS) agar plates (Liofilchem) and incubated at 37°C for 2 days. Distinct colonies were identified as *L. otakiensis*, L. rhamnosus, and *L. paracasei* by matrix-assisted laser desorption ionization–time-of-flight (MALDI-TOF) mass spectrometry using a Microflex LT instrument (Bruker Daltonics, Germany). Isolates were stored at −20°C in tryptone soya broth (Liofilchem) containing 15% glycerol. For whole-genome sequencing, isolates were grown in MRS broth at 37°C for 4 to 5 days, and the genomic DNA was extracted using the Quick-DNA fungi/bacterial kit (Zymo Research, USA), according to the manufacturer’s protocol. The Illumina Nextera XT DNA library preparation kit was used to prepare Illumina-specific libraries (8). Genome sequencing was performed using an Illumina NextSeq 500 sequencer (USA). Quality check of the single-end reads was performed using FastQC v.0.11.9 (https://www.bioinformatics.babraham.ac.uk/projects/fastqc/), and low-quality sequences and adaptors were removed using Cutadapt v.3.4 and fastp (9, 10). Then, the reads were corrected using Bloocoo (11). Default parameters were used unless otherwise specified. The quality-filtered reads were assembled *de novo* using SPAdes v.3.15.3, with error correction turned off, and MEGAHIT (12, 13), with automatic k-mer size selection. The assemblies were merged using GAM-NGS (14). The assembly quality was checked using BUSCO v.5.2.2 (15). Prokka (rapid prokaryotic genome annotation) was used for functional annotation (16). Information on the quality and genome features of the *de novo* assembly is presented in Table 1. Antimicrobial resistance (AMR) genes were predicted using the CARD Resistance Gene Identifier tool; AMR genes were not detectable with perfect or strict matches in our strains (17). Concatenated single-copy gene clusters of the novel and GenBank reference genomes were analyzed using Anvi’o (18), and the average nucleotide identity was calculated using pyANI (Fig. 1B) (19). The phylogenetic relationships were reconstructed using FastTree (Fig. 1A) (20). Both the pyANI and phylogenetic analyses suggested that the genomes described here grouped together and were closely related to reference sequences of the same LAB species.

**TABLE 1:** Quality information and genome features of the *de novo* assembled strains Lentilactobacillus otakiensis cek-R3, Lacticaseibacillus rhamnosus cek-R1, and Lacticaseibacillus paracasei subsp. *paracasei* cek-R2, originating from beetroot^*a*^

Strain	Total no. of reads	GenBank accession no.	SRA accession no.	Genome coverage (×)	No. of contigs	No. of coding sequences	No. of			Genome size (bp)	*N*_50_ (bp)	GC content (%)	BUSCOs (%)	% ANI (reference strain)
							rRNAs	tmRNAs	tRNAs					
cek-R1	7,250,258	JAIPUO000000000	SRS10102441	277	83	2,762	4	1	54	2,968,173	137,353	46.65	99.2	97.2 (L. rhamnosus)
cek-R2	6,996,786	JAIPUN000000000	SRS10102442	245	166	2,883	3	1	51	3,033,512	53,521	46.18	99.2	98.4 (*L. paracasei*)
cek-R3	4,084,504	JAIPUM000000000	SRS10102443	219	52	2,370	3	1	57	2,429,274	137,314	42.41	99.2	99.8 (*L. otakiensis*)

a tmRNAs, transfer-messenger RNAs; ANI, average nucleotide identity; BUSCOs, Benchmarking Universal Single-Copy Orthologs.

The features identified in the genomes of the described strains will assist us in better understanding their beneficial properties.

### Data availability.

The draft genome sequences of *Lacticaseibacillus* sp. strains cek-R1 and cek-R2 and *Lentilactobacillus* sp. strain cek-R3 have been deposited in GenBank under accession numbers JAIPUO000000000.1, JAIPUN000000000, and JAIPUM000000000, respectively. The raw reads can be found in the SRA under BioProject accession number PRJNA761968.
